# Cardiovascular outcomes following percutaneous coronary intervention with drug-eluting balloons in chronic kidney disease: a retrospective analysis

**DOI:** 10.1186/s12882-020-02089-w

**Published:** 2020-10-23

**Authors:** Michael Jonas, Maayan Kagan, Gal Sella, Dan Haberman, Gil Chernin

**Affiliations:** 1 Heart Institute, Kaplan Medical Center, Hebrew University School of Medicine, Rehovot, Israel; 2 Department of Nephrology and Hypertension, Kaplan Medical Center, Hebrew University School of Medicine, Pasternak St. POB1, 76100 Rehovot, Israel

**Keywords:** Drug-eluting stents, Chronic kidney disease, Drug-eluting balloons, Percutaneous coronary intervention, Dual anti-platelet therapy, Cardiovascular mortality, In-stent restenosis, Ischemic heart disease, Target lesion revascularization

## Abstract

**Background:**

Chronic kidney disease (CKD) is associated with poorer outcomes following percutaneous coronary intervention (PCI) with drug-eluting stents. Drug-eluting balloons are used for in-stent restenosis and selected cases of de-novo coronary lesions. Little is known regarding the outcomes of individuals with CKD who undergo PCI with drug-eluting balloons. The goal of this study was to assess outcomes of PCI with drug-eluting balloons in individuals with CKD.

**Methods:**

In a retrospective analysis, outcomes of PCI with drug-eluting balloons were compared between 101 patients with CKD and 261 without CKD. CKD was defined as estimated glomerular filtration rate < 60 ml/min/1.73m^2^. We compared demographics, procedure data and clinical outcomes in the first and second years following the procedure.

**Results:**

Rates of major adverse cardiac events (MACE) and myocardial infarction were higher in patients with than without CKD: 23.8% vs. 13.8%, *P* < 0.005 and 15.9% vs. 3.8%, *P* < 0.001, respectively. Rates of target lesion revascularization were similar, 14.9 and 11.5%, respectively, *P* = 0.4. Shorter duration of dual anti-platelet therapy was observed among patients with than without CKD (10.0 + 3.4 vs. 10.9 + 3.7 months, *P* < 0.05). First-year hemorrhage episodes were similar in the two groups (0.08 ± 0.4 and 0.03 ± 0.2, respectively, *P* = 0.2). In a multivariate regression analysis, CKD was associated with increased risks of first year MACE (OR 2.1; 95% confidence interval 1.0-4.3, *P* < 0.001).

**Conclusions:**

PCI with drug-eluting balloons was associated with increased cardiovascular morbidity and mortality in patients with than without CKD. However, rates of target lesion revascularization were similar in the two groups. Shorter duration of dual anti-platelet therapy was observed in the CKD group.

## Background

Percutaneous coronary intervention (PCI) with drug-eluting balloons (DEB) was established as an effective treatment for patients with ischemic heart disease (IHD) and in-stent restenosis (ISR) [1, 2]. In selected patients with IHD, DEBs are also used for de-novo lesions, though small randomized control trials reported conflicting results in this regard [1, 3, 4]. DEBs mechanically open obstructed vessels by inflating; this releases a homogeneous high dose of anti-proliferative drug (such as paclitaxel). The resultant reduction in neointimal proliferation prevents restenosis of the obstructed vessel [2, 5].

Unlike the commonly used drug-eluting stents (DES), DEB angioplasty does not entail implantation of a foreign body in the blood vessel. Thus, a shorter period of dual anti-platelet therapy (DAPT) is required, since it is conceivable that DEB does not confer a risk for stent thrombosis. A shorter period of DAPT could be beneficial for patients with bleeding diathesis, such as patients with chronic kidney disease (CKD) [2, 6].

Renal dysfunction is a known risk factor for IHD, and is associated with accelerated atherosclerosis, endothelial dysfunction, oxidative stress and inflammation. These contribute to the formation of coronary artery atherosclerosis and increased cardiovascular mortality [7–9]. In addition, individuals with CKD who underwent PCI with DES were shown to have poorer post-procedural cardiovascular outcomes than patients without CKD, including higher mortality, hemorrhagic complications and prolonged hospitalizations [9, 10].

Little is known about DEB angioplasty outcomes among patients with CKD. Data from the Korean Multicenter In-Stent Restenosis Registry suggest that while rates of target lesion failure were lower in contemporary DES compared with DEB, this difference was attenuated in patients with CKD (defined as an estimated glomerular filtration rate (eGFR) < 60/ml/min/1.73 m2) [11]. The aim of the current study was to compare outcomes of patients who underwent PCI with DEB, between those with and without CKD.

## Methods

This study was approved by the Institutional Review Board of the Kaplan Medical Center. We performed a retrospective, all comers, single center registry of patients who underwent DEB angioplasty between August 2011 and October 2017. Electronic medical records of enrolled patients were obtained from the electronic system of the Kaplan Medical Center and from the ‘Ofek’ system, the medical record system of the Clalit Health Services, the largest health provider in Israel.

Patients meeting the inclusion criteria were males and non-pregnant females, older than 18 years, who attended regular follow-up with cardiology consultants at least every 6 months after the procedure, and who had at least two creatinine serum level tests. The first of these tests was three to 6 months prior to the procedure, and the second test in the 3 months before the PCI with DEB angioplasty. Clinical indications for angiography included acute coronary syndrome (ACS), angina pectoris, congestive heart failure (CHF) or arrythmia evaluation, or a positive stress test. Patients with acute kidney injury, as defined by the KDIGO criteria [12], or a recent myocardial infarction (MI) defined as ≤ 7 days of PCI were excluded from the analysis. The decision to use DEB was made by individual operators under considerations of lesion complexity, the inability to deliver a stent or the requirement for a shorter DAPT regimen (either due to bleeding tendencies or the need to temporarily stop DAPT for planned non-vascular invasive procedures).

For each patient who met the eligibility criteria we reviewed baseline characteristics including age, gender, body mass index and ejection fraction measured in the year before the PCI. Additionally, we reviewed background conditions such as CHF, diabetes mellitus, chronic obstructive pulmonary disease, hypertension and hyperlipidemia.

### Definition of variables, measurements and procedure characteristics

Creatinine baseline was defined as the closest measurement prior to the procedure, within 3 months. For each patient, we used the baseline measurement to calculate eGFR with the CKD-EPI formula [13]. Creatinine assay in this report used methods that are traceable to isotope dilution mass spectrometry (IDMS) reference methodology. The cohort was stratified according to the absence or presence of CKD, defined as eGFR < 60/ml/min/1.73m^2^ for more than 3 months. The eGFR was calculated according to the last creatinine before the PCI “.

We assessed the characteristics of the angioplasty including the indication of the catheterization (ISR or de-novo), the target vessel (right coronary artery, left main coronary artery, left anterior descending artery, left circumflex or graft vessel), the type of DEB used in the procedure, the number of balloons used during the procedure, the length and size of the lesion, the type of DAPT that was recommended following the procedure (Clopidogrel, Ticagrelor or Prasugrel) and the duration of DAPT following the procedure. The duration of DAPT was analyzed both continuously and categorically (categories were defined as below or above 6 months).

The DEBs that were used in the procedures, at a dose of 3.0 μg/mm^2^ of paclitaxel, were ‘Pantera Lux’ (Biotronik, Bülach, Switzerland) or ‘SeQuent Please’ (B. Braun, Melsungen AG, Berlin, Germany) or both. Additionally, some of the procedures used ‘AngioSculpt’ by Biotronik.

Seventeen patients underwent two procedures during the registry years. For each of these patients, we analyzed data from the first procedure and noted the additional procedure as an outcome.

For twelve patients, an intervention was performed in more than one vessel during the PCI. We excluded these patients from the analysis of vessel type, lesion length and lesion size. These parameters were included in the regression analysis.

### Outcomes

The main outcome was major adverse cardiac event (MACE) during the first year, defined as the composite of three endpoints: cardiac hospitalizations, cardiac death and target lesion revascularization (TLR). Cardiac hospitalizations were defined as hospitalizations due to one of the following: MI, ACS, chest pain (without an alternative non-cardiac diagnosis), arrhythmia or heart failure exacerbation. Cardiac death was defined as death due to one of the above cardiac hospitalization etiologies. Additional analyses of first and second-year cardiac death and cardiac hospitalizations were obtained. We also analyzed first year hemorrhages, defined as type 2 or higher bleeding events according to the BARC criteria [14].

### Statistical analysis

Continuous variables were described using means ± standard deviations; categorical variables were described by frequencies and percentages. The Chi square test, T test and Fisher’s exact test were used to calculate differences between the groups, as appropriate. A multivariant regression analysis was used to determine the independent effect of CKD on the outcomes examined. The Kaplan-Meier plot was used to estimate survival in the first year of follow up using the log-rank test. All statistical analyses were performed with SAS version 9.4 (SAS Institute, Cary, North Carolina).

## Results

Of 362 patients who met the inclusion criteria, 101 had CKD, defined as baseline eGFR< 60 ml/min/1.73m^2^. Baseline characteristics are summarized in Table 1. Two patients were excluded from the analysis because of lack of first creatinine levels three to 6 months prior to the procedure. The mean eGFR for the CKD and non-CKD groups were 40.2 ± 17.9 and 86.1 ± 17.4 ml/min/1.73m^2^, respectively. Of the 101 patients with CKD, 52 had CKD stage 3A (eGFR 45-59 ml/min/1.73 m2), 25 had CKD stage 3B (eGFR 30-44 ml/min/1.73 m2), and 24 had CKD stage 4 (eGFR 15-29 ml/min/1.73 m2).

**Table 1:** Baseline characteristics of patients who underwent percutaneous coronary intervention with drug-eluting balloons

	Full cohort				In-stent restenosis				De-novo lesions			
	All patients	Non-CKD	CKD	***P*** value	All patients	Non-CKD	CKD	***P*** value	All patients	Non-CKD	CKD	***P*** value
**Patients (n)**	362	261	101		101	65	36		261	196	65	
**eGFR (ml/min/1.73**^**2**^**)**	73.2 ± 27.1	86.1 ± 17.4	40.2 ± 17.9	**< 0.001**	70.2 ± 27.5	86.5 ± 16.4	40.7 ± 17.2	**< 0.001**	74.5 ± 26.8	86.0 ± 17.8	39.9 ± 18.4	**< 0.001**
**Men (%)**	291 (80.4%)	219 (83.9%)	72 (71.3%)	**< 0.01**	75 (74.3%)	51 (78.5%)	24 (66.7%)	0.2	216 (82.8%)	168 (85.7%)	48 (73.9%)	**< 0.05**
**Age (years)**	66.4 ± 11.4	64.0 ± 10.8	72.5 ± 10.8	**< 0.001**	67.4 ± 10.9	64.0 ± 9.7	73.5 ± 10.2	**< 0.001**	66.0 ± 11.6	64.0 ± 11.2	71.9 ± 11.1	**0.001**
**BMI (%)**	28.6 ± 4.5	28.4 ± 4.5	28.9 ± 4.5	0.4	27.7 ± 4.0	27.8 ± 4.1	27.6 ± 3.7	0.8	28.9 ± 4.6	28.6 ± 4.6	29.7 ± 4.8	0.1
**Background condition**												
**Smoker (%)**	140 (38.7%)	112 (42.9%)	28 (27.7%)	**< 0.01**	38 (37.6%)	27 (41.5%)	11 (30.6%)	0.3	102 (39.1%)	85 (43.4%)	17 (26.2%)	**< 0.05**
**Diabetes mellitus (%)**	166 (45.9%)	105 (40.2%)	61 (60.4%)	**< 0.001**	55 (54.5%)	31 (47.7%)	24 (66.7%)	0.1	111 (42.5%)	74 (37.8%)	37 (56.9%)	**< 0.01**
**COPD (%)**	22 (6.1%)	11 (4.2%)	11 (10.9%)	**< 0.05**	5 (5.0%)	3 (4.6%)	2 (5.6%)	1	17 (6.5%)	8 (4.1%)	9 (13.9%)	**< 0.05**
**Hyperlipidemia (%)**	259 (71.6%)	191 (73.2%)	68 (67.3%)	0.3	79 (78.2%)	53 (81.5%)	26 (72.2%)	0.3	180 (69.0%)	138 (70.4%)	42 (64.6%)	0.4
**Hypertension (%)**	263 (72.8%)	173 (66.5%)	90 (89.1%)	**< 0.001**	77 (76.2%)	47 (72.3%)	30 (83.3%)	0.2	186 (71.5%)	126 (64.6%)	60 (92.3%)	**< 0.001**
**ACS (%)**	139 (38.4%)	92 (35.3%)	47 (46.5%)	**0.05**	35 (34.7%)	19 (29.2%)	16 (44.4%)	0.1	104 (39.9%)	73 (37.2%)	31 (47.7%)	0.2
**CHF (%)**	18 (5.0%)	4 (1.5%)	14 (13.9%)	**< 0.001**	10 (9.9%)	2 (3.1%)	8 (22.2%)	**< 0.005**	8 (3.1%)	2 (1.0%)	6 (9.2%)	**< 0.005**
**EF (%)**	48.2 ± 8.8	49.4 ± 8.0	45.0 ± 10.0	**< 0.001**	45.7 ± 9.3	47.5 ± 8.6	42.6 ± 9.9	**< 0.05**	49.1 ± 8.4	50.0 ± 7.7	46.4 ± 9.8	**< 0.01**

*ACS* Acute coronary syndrome, *BMI* Body mass index, *CHF* Chronic heart failure, *COPD* Chronic obstructive pulmonary disease, *CKD* Chronic kidney disease, *EF* Ejection fraction, *eGFR* Estimated glomerular filtration rate

Compared to patients without CKD, among those with CKD, the mean age was older (72.5 + 10.8 versus 64.0 + 10.8 years, *P < 0.001)* and the proportion of males was smaller (71.3% vs. 83.9%, *P < 0.01).* Additionally, the CKD group had substantially higher rates of comorbidities such as diabetes mellitus (60.4% vs. 40.2%, *P* < 0.001), hypertension (89.1% vs. 66.5%, *P <* 0.001) and CHF (13.9% vs. 1.5%, *P < 0.001)*; and a lower rate of smoking (27.7% vs. 42.9%, *P < 0.01*) (Table 1).

Of 261 patients with PCI with DEB due to de-novo lesions, 65 (25%) had CKD (Table 1). Compared to patients without CKD, those with CKD were older and more likely with diabetes, CHF and hypertension; and less likely smokers.

Of the 101 patients who underwent PCI with DEB due to ISR, 97 had restenosis in DES, 3 patients in bare metal stents, and the type of stent was unknown in one patient. Thirty-six (36%) of these patients had CKD. Compared to those without CKD, the mean age of those with CKD was older (Table 1), and the proportion with CHF was higher.

### Procedure characteristics, lesion morphology and antiplatelet therapy

Angiographic data and procedure characteristics are presented in Table 2. De-novo catheterization, compared to ISR, was the indication for PCI in a lower proportion in the CKD than the non-CKD group (64.4% vs. 75.1%, *P < 0.05).* The proportions of procedures that were ambulatory rather than urgent were similar between the groups. Lesion size and length were similar for the CKD group (mean 2.7 ± 0.6 mm and 22.5 ± 6.4 mm, respectively) and the non-CKD group (mean 2.6 ± 0.6 mm and 22.7 ± 6.5 mm, respectively). The number of balloons used was similar in the two groups (mean 1.1 ± 0.4 and 1.1 ± 0.2, respectively, *P = 0.1*) (Table 2). The types of DEB (‘Pantera Lux’ vs. ‘SeQuent Please’ vs. both) did not differ significantly, according to the presence of CKD. The usage of both DEBs vs. Pantera Lux alone was higher for the CKD than the non-CKD group (15.5% vs. 7.5%, *P < 0.05).*

**Table 2:** Procedure characteristics of patients who underwent percutaneous coronary intervention with drug-eluting balloons

	Full cohort				In-stent restenosis				De-novo lesions			
Variable	All patients	Non-CKD	CKD	***P*** value	All patients	Non-CKD	CKD	***P*** value	All patients	Non-CKD	CKD	***P*** value
**Patient (n)**	362	261	101		101	65	36		261	196	65	
**Indication**				***< 0.05***								
***Restenosis*** **(%)**	101 (27.9%)	65 (24.9%)	36 (35.6%)									
**De novo (%)**	261 (72.1%)	196 (75.1%)	65 (64.4%)									
**ACS (%)**	238 (65.8%)	174 (66.7%)	64 (63.4%)	0.6	59 (58.4%)	34 (52.3%)	25 (69.4%)	0.1	179 (68.6%)	140 (71.4%)	39 (60.0%)	0.1
**Target vessel**				0.3				0.2				0.1
**LCX (%)**	134 (38.3%)	95 (37.3%)	39 (41.0%)		29 (29.9%)	22 (34.9%)	7 (20.6%)		105 (41.5%)	73 (38.0%)	32 (52.5%)	
**LAD (%)**	147 (42.0%)	113 (44.3%)	34 (35.8%)		41 (42.3%)	24 (38.1%)	17 (50.0%)		106 (41.9%)	89 (46.4%)	17 (27.9%)	
**LMCA (%)**	5 (1.4%)	2 (0.8%)	3 (3.2%)		4 (4.1%)	1 (1.6%)	3 (8.8%)		1 (0.4%)	1 (0.5%)	0 (0%)	
**RCA (%)**	59 (16.9%)	42 (16.5%)	17 (17.9%)		18 (18.6%)	13 (20.6%)	5 (14.7%)		41 (16.2%)	29 (15.1%)	12 (19.7%)	
**Graft (%)**	5 (1.4%)	3 (1.2%)	2 (2.1%)		5 (5.2%)	3 (4.8%)	2 (5.9%)		0 (0%)	0 (0%)	0 (0%)	
**DEB- type**				0.1				0.6				***0.01***
***Pantera Lux*** **(%)**	160 (45.6%)	120 (47.2%)	40 (41.2%)		40 (41.2%)	28 (44.4%)	12 (35.3%)		120 (47.2%)	92 (48.2%)	28 (44.4%)	
***SeQuent Please*** **(%)**	157 (44.7%)	115 (45.3%)	42 (43.3%)		50 (51.6%)	30 (47.6%)	20 (58.8%)		107 (42.1%)	85 (44.5%)	22 (34.9%)	
***Both*** **(%)**	34 (9.7%)	19 (7.5%)	15 (15.5%)		7 (7.2%)	5 (7.9%)	2 (5.9%)		27 (10.6%)	14 (7.3%)	13 (20.6%)	
**Number of balloons**	1.1 ± 0.3	1.1 ± 0.2	1.1 ± 0.4	0.1	1.1 ± 0.3	1.1 ± 0.3	1.1 ± 0.3	0.9	1.1 ± 0.3	1.0 ± 0.2	1.1 ± 0.4	0.1
**Size**	2.6 ± 0.6	2.6 ± 0.6	2.7 ± 0.6	0.5	3.0 ± 0.6	3.0 ± 0.6	3.0 ± 0.6	0.7	2.5 ± 0.5	2.5 ± 0.5	2.5 ± 0.5	0.9
**Lesion length (mm)**	22.6 ± 6.5	22.7 ± 6.5	22.5 ± 6.4	0.8	22.3 ± 6.1	22.3 ± 6.1	22.5 ± 6.3	0.9	22.7 ± 6.6	22.8 ± 6.7	22.4 ± 6.5	0.7
**DAPT- type**				***< 0.01***				1				***< 0.005***
**Prasugrel or Ticagralor (%)**	87 (24.0%)	73 (28.0%)	14 (13.9%)		14 (13.9%)	9 (13.9%)	5 (13.9%)		73 (28.0%)	64 (32.7%)	9 (13.9%)	
**Clopidogral (%)**	275 (76.0%)	188 (72.0%)	87 (86.1%)		87 (86.1%)	56 (86.2%)	31 (86.1%)		188 (72.0%)	132 (67.4%)	56 (86.2%)	
**DAPT duration- continues, months**												
***All***	10.6 ± 3.7	10.9 ± 3.7	10.0 ± 3.4	***< 0.05***	10.2 ± 3.3	10.4 ± 3.2	10.1 ± 3.4	0.8	10.8 ± 3.8	11.1 ± 3.8	9.8 ± 3.5	***< 0.005***
***Ambulatory cohort***	10.8 ± 3.6	11.3 ± 4.6	9.6 ± 3.3	***< 0.05***	10.0 ± 3.3	9.8 ± 3.3	10.4 ± 3.6	0.7	11.2 ± 4.8	12.1 ± 5.0	9.2 ± 3.8	0.01
**DAPT duration- categoric, < 6 month (%)**	54 (15.0%)	33 (12.7%)	21 (20.8%)	0.1	18 (17.8%)	12 (18.5%)	6 (16.7%)	1	36 (13.9%)	21 (10.8%)	15 (23.1%)	***< 0.05***

*ACS* Acute coronary syndrome, *DEB* Drug eluting balloon, *DAPT* Dual anti platelet therapy, *eGFR* Estimated glomerular filtration rate, *LAD* Left anterior descending, *LCX* Left circumflex, *LMCA* Left main coronary artery, *RCA* Right coronary artery

For the patients with CKD, DAPT therapy with Prasugrel or Ticagrelor was less often implemented than was DAPT with Clopidogrel (76.0% vs. 24.0%, *P < 0.01)*. Shorter duration of DAPT was observed among patients with than without CKD (10.0 ± 3.4 vs. 10.9 ± 3.7 months, *P* < 0.05*).* Among those who underwent an ambulatory procedure, the duration of DAPT was shorter for those with than without CKD (9.6 ± 3.3 vs. 11.3 ± 4.6 months, *P < 0.05).*

In the sub-analysis of the de-novo cohort, lesion size and length were similar between those with and without CKD. The recommendation of DAPT with Prasugrel or Ticagrelor was substantially lower for patients with than without CKD (13.9% vs. 32.7%*, P < 0.005)*, and the usage of both DEBs, vs. Pantera lux alone or vs. SeQuent Please alone, was greater (20.6% vs. 7.3%, *P = 0.01)*. The duration of DAPT was shorter for the CKD group (9.8 ± 3.5 vs. 11.1 ± 3.8, *P < 0.005)* (Table 2). In the ISR cohort, lesion size and length, DAPT type, DAPT duration and DEB type were similar between the patients with and without CKD (Table 2).

### Outcomes

**Fig. 1:**
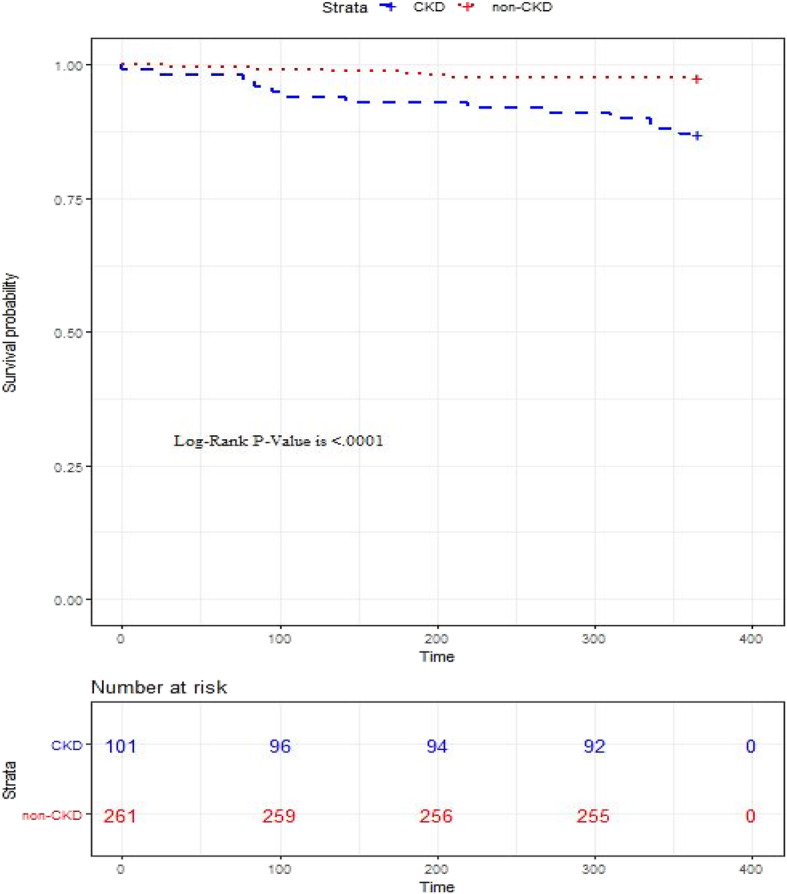
Kaplan-Meier plot for one-year survival following percutaneous coronary interventions with drug-eluting balloons. CKD, Chronic Kidney Disease

Outcomes for the first and second years of follow up are summarized in Tables 3 and 4, and Fig. 1. MACE rates after 1 year were significantly higher for the CKD group (23.8% vs. 13.8%, *P <* 0.005). Compared to patients without CKD, for those with CKD, rates were higher of first year MI (15.9% vs. 3.8%, *P < 0.001*) and all-cause death (12.9% vs. 2.3%, *P < 0.001*) (Fig. 1). Moreover, the CKD group had significantly higher rates of all-cause hospitalizations during the first month (0.3 ± 1.0 vs. 0.1 ± 0.4, *P < 0.05*) and first year (1.9 ± 3.0 vs. 0.8 ± 1.4*, P = 0.001*). Cardiac hospitalizations were also more common in the CKD group during the first year (1.1 ± 1.7 vs. 0.5 ± 1.0, *P < 0.005*) (Table 3). The groups were comparable for first year TLR, cerebrovascular accidents and major hemorrhagic events (Table 3).

**Table 3:** Outcomes following DEB of patients who underwent percutaneous coronary intervention with drug-eluting balloons

	Full cohort				In-stent restenosis				De-novo lesions			
Variable	All patients	Non-CKD	CKD	***P*** value	All patients	Non-CKD	CKD	***P*** value	All patients	Non-CKD	CKD	***P*** value
**Patients (n)**	362	261	101		101	65	36		261	196	65	
**MACE- 1st year (%)**	60 (16.6%)	36 (13.8%)	24 (23.8%)	**< 0.05**	25 (24.8%)	11 (16.9%)	14 (38.9%)	**< 0.05**	35 (13.4%)	25 (12.8%)	10 (15.4%)	0.7
**MI- 1st year (%)**	26 (7.2%)	10 (3.8%)	16 (15.9%)	**< 0.001**	16 (15.85%)	5 (7.7%)	11 (30.6%)	**< 0.01**	10 (3.8%)	5 (2.6%)	5 (7.7%)	0.1
**Death- 1st year (%)**	19 (5.3%)	6 (2.3%)	13 (12.9%)	**< 0.001**	8 (7.9%)	0 (0%)	8 (22.2%)	**< 0.001**	11 (4.2%)	6 (3.1%)	5 (7.7%)	0.2
**Death- 2nd year (%)**	35 (9.7%)	12 (4.6%)	23 (22.8%)	**< 0.001**	13 (12.9%)	1 (1.5%)	12 (33.3%)	**< 0.001**	22 (8.4%)	11 (5.6%)	11 (16.9%)	**< 0.01**
**Cardiac death- 1st year (%)**	4 (1.1%)	1 (0.4%)	3 (3.0%)	0.1	2 (2.0%)	0 (0%)	2 (5.6%)	0.1	2 (0.8%)	1 (0.5%)	1 (1.5%)	0.4
**Cardiac death- 1st + 2nd years (%)**	8 (2.2%)	2 (0.8%)	6 (5.9%)	**< 0.01**	4 (4.0%)	1 (1.5%)	3 (8.3%)	0.1	4 (1.5%)	1 (0.5%)	3 (4.6%)	**< 0.05**
**Hospitalizations- 1st month**	0.2 ± 0.6	0.1 ± 0.4	0.3 ± 1.0	**< 0.05**	0.3 ± 0.9	0.2 ± 0.4	0.4 ± 1.4	0.2	0.2 ± 0.4	0.1 ± 0.3	0.3 ± 0.6	**< 0.05**
**Hospitalizations- 1st year**	1.1 ± 2.0	0.8 ± 1.4	1.9 ± 3.0	**0.001**	1.5 ± 3.1	0.8 ± 1.6	2.8 ± 4.5	**< 0.05**	1.0 + 1.4	0.9 ± 1.3	1.4 ± 1.7	**< 0.05**
**Hospitalizations- 1st + 2nd year**	1.7± 2.7	1.3 ± 1.9	2.8 ± 4.0	**< 0.001**	2.3 ± 4.0	1.6 ± 2.2	3.9 ± 5.8	**< 0.05**	1.5 ± 2.0	1.2 ± 1.8	2.3 ± 2.3	**< 0.01**
**Cardiac hospitalizations- 1st year**	0.7 ± 1.2	0.5 ± 1.0	1.1 ± 1.7	**< 0.005**	0.9 + 1.7	0.6 ± 1.3	1.5 ± 2.2	**< 0.05**	0.6 ± 0.9	0.5 ± 0.8	0.8 ± 1.2	**< 0.05**
**Cardiac hospitalizations- 1st + 2nd years**	1.0 ± 1.7	0.8 ± 1.3	1.6 ± 2.3	**< 0.001**	1.5 + 2.3	1.2 ± 1.8	2.2 ± 2.9	0.1	0.8 ± 1.3	0.7 ± 1.1	1.3 ± 1.7	**< 0.01**
**1st Year catheterization**	0.3± 0.6	0.4 ± 0.6	0.3 ± 0.5	0.4	0.3 + 0.6	0.3 ± 0.6	0.3 ± 0.5	1	0.3 ± 0.6	0.4 ± 0.6	0.3 ± 0.5	0.4
**TLR (%)**	45 (12.4%)	30 (11.5%)	15 (14.9%)	0.4	15 (14.9%)	8 (12.3%)	7 (19.4%)	0.4	30 (11.5%)	22 (11.2%)	8 (12.3%)	0.8
**Additional DEB (%)**	17 (4.7%)	14 (5.4%)	3 (3.0%)	0.4	6 (5.9%)	4 (6.2%)	2 (5.6%)	1	11 (4.2%)	10 (5.1%)	1 (1.5%)	0.3
**Cerebrovascular accident- 1st year (%)**	7 (2.0%)	4 (1.5%)	3 (3.0%)	0.4	2 (2.0%)	1 (1.5%)	1 (2.8%)	1	5 (1.9%)	3 (1.5%)	2 (3.1%)	0.6
**Hemorrhages- 1st year**	0.05 ± 0.3	0.03 ± 0.2	0.08 ± 0.4	0.2	0.05 ± 0.3	0.03 ± 0.2	0.08 ± 0.4	0.4	0.05 ± 0.2	0.04 ± 0.2	0.08 ± 0.3	0.3

*DEB* Drug eluting balloon, *eGFR* Estimated glomerular filtration rate, *MACE* Major adverse cardiac events, *MI* Myocardial infarction, *TLR* Target lesion revascularization

**Table 4:** Regression analysis of the independent effect of CKD on outcomes

Variable	Coefficient	CI 95%	SD	OR	OR CI 95%	RR	RR CI 95%	***P*** value
**MACE-- 1st year**	0.7	0.02 – 1.5	0.4	2.1	1.0 - 4.3	1.8	0.9 – 3.3	**< 0.05**
**MI- 1st year**	1.6	0.5 – 2.6	0.5	4.8	1.7 – 14.2	3.6	1.4 – 9.1	**< 0.005**
**Death- 1st year**	1.1	(−0.2) - 2.4	0.7	3	0.9 - 11.1	2.6	0.8 – 8.3	0.1
**Death- 2nd year**	0.9	0.03 - 1.9	0.5	2.6	3.6 - 6.7	2.2	0.9 - 5	**< 0.05**
**Cardiac death- 1st year**	41.3	(−33,096) - 33,178.9	16,845.8	0	0-inf	0	0-inf	1
**Cardiac death- 1st + 2nd years**	0.8	(−1.3) - 3.0	1.1	2.3	0.3 - 20.0	1.9	0.3 – 12.5	0.5
**Hospitalizations- 1st year**	0.7	0.2 - 1.2	0.3					**0.01**
**Hospitalizations- 1st + 2nd year**	1.1	0.4 - 1.8	0.4					**< 0.05**
**Cardiac hospitalizations- 1st year**	0.5	0.1 - 0.8	0.2					**< 0.01**
**Cardiac hospitalizations- 1st + 2nd years**	0.7	0.3 - 1.2	0.2					**0.001**
**1st year catheterization**	0.01	(−0.2) - 0.2	0.08					0.9
**TLR**	0.6	(−0.2) - 1.4	0.4	1.8	0.8 - 4	1.6	0.8 – 3.4	0.2
**Additional DEB**	(−1.2)	(−2.9) - 0.5	0.9	0.3	0.1 - 1.6	0.3	0.1 – 1.6	0.2
**Stroke- 1st year**	0.3	(−2.0) - 2.7	1.2	1.4	0.1 – 14.3	1.4	0.1 – 14.2	0.8
**Hemorrhages- 1st year**	0.02	(−0.05) -0.09	0.04					0.6

*DEB* Drug eluting balloon, *MACE* Major adverse cardiac events, *MI* Myocardial infraction, *TLR* Target lesion revascularization

Among patients who underwent PCI with DEB for ISR, higher rates of MACE (38.9% vs. 16.9%, *P < 0.05*) and MI (30.6% vs. 7.7%, *P < 0.01*) were noted in those with than without non-CKD (Table 3**)**. Among patients who underwent PCI with DEB for de-novo lesions, rates of MACE and MI were not statistically different between CKD and non-CKD patients (Table 3).

In a multivariate regression model (linear and logistic, as appropriate), CKD was associated with increased risks of first year MACE (OR 2.1; 95% confidence interval 1.0- 4.3, *P* < 0.001) and MI (OR 4.8; 95% confidence interval 1.7- 14.2, *P* < 0.005) (Table 4).

## Discussion

In this study of patients who underwent PCI with DEB, rates of MACE, MI, cardiac and all-cause hospitalizations, and cardiac and all-cause death were higher among those with CKD, defined as eGFR< 60 ml/min/1.73m^2^. To the best of our knowledge, this observational study is the first to thoroughly detail the outcomes of patients with CKD who underwent PCI with DEB. Nguyen et al found an association of CKD with increased MACE in patients treated with DEB for ISR. Yet, that study comprised a small number of patients with CKD, and CKD was not clearly defined [15].

Patients with CKD in the current study were older and had more risk factors associated with cardiac events and mortality, such as hypertension, heart failure and diabetes mellitus. Indeed, in this report, the increased age and the traditional risk factors contributed significantly to the adverse cardiovascular outcomes observed in the patients with CKD. In the multivariate regression analysis, CKD was significantly associated with cardiac hospitalizations, all-cause hospitalizations, and first and second-year all-cause death. Surprisingly, despite the poorer cardiovascular outcomes in the CKD group, the rates of TLR were similar for those with and without CKD. Due to the retrospective study design, we can only speculate regarding the underlying mechanisms leading to these rates. One possible explanation is that patients with CKD do not have higher rates of restenosis; this would imply that higher rates of cardiac events occur due to other mechanisms associated with CKD, such as arrhythmias, cardiac remodeling and heart failure [16]. These mechanisms are consequent to such changes as chronic volume overload, hyperactivation of the renin-angiotensin system and erythropoietin deficiency anemia [16, 17]. Another explanation for the similarity in TLR rates is physician’s reluctance to catheterize patients with CKD due to the increased risk for complications following PCI [18, 19]. Lower rates of catheterization could lead to both underestimation of lesion restenosis and poorer cardiovascular outcomes.

The association between CKD and cardiovascular disease is well established [16]. Indeed, cardiovascular disease is the most common cause of morbidity and mortality among CKD patients [16, 17, 20, 21]. CKD patients are more prone to IHD and tend to have poorer outcomes following revascularization with PCI or coronary artery bypass surgery [8, 22]. Even in the era of PCI with the newer generation of DES, and with well-balanced guideline-based medical therapy, adverse cardiovascular outcomes are more frequent in those with than without CKD [10, 19, 23]. Additionally, higher rates of MACE were recently reported in patients on hemodialysis who underwent PCI with DEB compared to patients not on hemodialysis [24]. Similarly, that study suggests an association of CKD with poorer outcomes following DEB angioplasty. However, in light of the potential benefits of DEB angioplasty for patients with CKD, outcomes of PCI with DEB versus DES should be investigated further, in selected coronary lesions.

Hypothetically, DEB angioplasty could be beneficial compared with the commonly used DES, for patients with a bleeding diathesis, such as those with CKD. This is because DEB enables a shorter period of DAPT, as short as 1-3 months (compared with the 12 month recommendation following DES implantation) [2, 6]. The current study showed a significantly shorter period of DAPT for the CKD group. However, while the minimal recommended time following DEB angioplasty is 1-3 months, the mean DAPT duration for the CKD group was 10.0 ± 3.4 months, suggesting that the full potential benefits of shorter DAPT was not realized. The DAPT recommendation following ACS is 12 months [5, 6]. Thus, we performed an additional analysis of the patients who underwent an ambulatory procedure, to assess physicians’ recommendation regarding DAPT, without the impact of ACS, which requires longer therapy. As expected, the DAPT duration was shorter for the CKD than the non-CKD group, yet still longer than the 1-3 months minimal required period. Furthermore, the difference between the CKD and the non-CKD groups in the ambulatory cohort was more substantial than in the full cohort. Interestingly, though the DAPT duration was longer than the minimally required duration, bleeding events were similar between the CKD and the non-CKD groups. Indeed, CKD patients may have a disrupted coagulation system and are prone to bleed following PCI with DAPT [22, 24]. These findings suggest that the shorter period of DAPT for the CKD group, though not fully exploited, may reduce bleeding events that could be life threatening [7]. Concurring with other studies, the rate of revascularization was lower among patients with than without CKD. One possible reason for such is the reluctance of physicians to catheterize patients with CKD due to complications such as bleeding [18, 22]. The option of shortening the DAPT duration should decrease concerns regarding long-term hemorrhagic events. Furthermore, recent studies showed that physician-guided cessation of DAPT after PCI with stenting is more frequent among patients with CKD, and this was shown to be associated with a higher rate of MACE [7]. Taken together, reducing the possible bleeding risk with shorter DAPT in patients with CKD may come at the expense of an increased risk for thrombotic events [7, 25]. Therefore, randomized clinical trials are needed of patients with CKD, to address the minimal DAPT duration required following PCI.

This study has several limitations that should be acknowledged. First and foremost, as in any retrospective study, associations between CKD and poor outcomes following DEB angioplasty do not indicate causality. CKD status was defined by eGFR, based on creatinine measurements in the 3 months before PCI. It is therefore possible that some patients without CKD developed CKD in this interim and some patients with CKD may have experienced worsening of their disease. The decision to use DEB was made by the operator and as such, the probability of major selection bias exists. Intravascular ultrasound (IVUS) was not used routinely but rather in selected cases. Therefore, additional data of the detection by IVUS of coronary artery morphology is lacking. Additionally, while DAPT duration was shorter in the CKD patients, the observed duration was still longer than the minimal required time [2]. Indeed, not all patients with CKD had a shorter duration of DAPT and this may reflect other considerations for prolonged DAPT (e.g. concomitant peripheral vascular disease).

## Conclusions

CKD was associated with adverse cardiovascular outcomes following PCI with DEB. Rates of TLR were similar for patients with and without CKD. Additional studies are needed to compare PCI with DEB versus DES, in the context of CKD, for the implementation of patient-tailored clinical recommendations.

## Data Availability

The datasets generated and analyzed for the current study are not publicly available due to institute regulations, but they are available from the corresponding author on reasonable request.

## Abbreviations

ACS: Acute coronary syndrome
CHF: Congestive heart failure
CKD: Chronic kidney disease
DAPT: Dual anti-platelet therapy
DES: Drug-eluting stents
eGFR: Estimated glomerular filtration rate
IHD: Ischemic heart disease
ISR: In-stent restenosis
MACE: Major adverse cardiac event
PCI: Percutaneous coronary intervention
TLR: Target lesion revascularization
